# A Comparison of Healthy Infants and Adults with Respect to Indirect Microparticle Activity and the Parameters of the Thrombin Generation Test

**DOI:** 10.4274/tjh.2015.0341

**Published:** 2016-05-16

**Authors:** Filiz Şimşek Orhon, Nejat Akar, Yonca Eğin, Betül Ulukol, Sevgi Başkan

**Affiliations:** 1 Ankara University Faculty of Medicine, Department of Pediatrics, Divisions of Social Pediatrics, Ankara, Turkey; 2 Ankara University Faculty of Medicine, Department of Pediatrics, Divisions of Pediatric Molecular Genetics, Ankara, Turkey

**Keywords:** infant, Adult, Microparticle, Thrombin

## TO THE EDITOR

Microparticles express phospholipids and support thrombin generation, which increases with age [1,2].

In a recently published study, we showed age-dependent changes in thrombin generation parameters in a healthy infant population aged 1-24 months [3]. The aim of this present study was to compare the levels of both indirect microparticle activity and thrombin generation parameters of healthy infants from our recent study to those of a healthy adult population. The adult population consisted of medical students of the Ankara University School of Medicine. Blood was collected into tubes containing 1 mL of 0.109 M trisodium citrate. For indirect microparticle activity, plasma samples were studied using the STA-PROCOAG-PPL Kit (Diagnostica Stago Inc., Asnières sur Seine, France). Plasma samples were measured using thrombin generation kits, including a Thrombin Calibrator, PPP-Reagent 5 pM, and the FluCa-Kit (Diagnostica Stago). Thrombin generation curves were calculated using Thrombinoscope software (Thrombinoscope BV, Maastricht, the Netherlands). The following parameters were derived from the curves: lag time (LT, min), time to initiation of thrombin generation; endogenous thrombin potential (ETP, nmol/L/min), area under the thrombin generation curve; peak thrombin activity (peak, nmol/L); and time to peak thrombin generated (TTP, min). Statistical analysis was performed using Statistical Package for the Social Sciences 16.

A total of 58 healthy adults (23 males and 35 females; mean age: 23.2±0.4 years) were admitted to the study. In our recent study, 85 healthy infants (51 males and 34 females; mean age: 12.6±8.3 months) were studied. The indirect microparticle activity in the infant group was significantly lower than that of the adult group (p<0.001). The ETP and peak levels in the infant group were significantly lower than those of adults. Furthermore, the TTP levels of the adult group were lower than those of infants (p=0.001) (Table 1).

Physiologic concentrations of coagulation proteins gradually increase after birth [4]. Karlaftis et al. showed that procoagulant phospholipid activity was increased in neonates and decreased in children aged 1-16 years [5]. We show that the levels of indirect microparticle activity are increased in healthy adults as compared to healthy infants. This may suggest that aging is correlated to an increase in the indirect microparticle activity, and also possibly to its procoagulant and proinflammatory features.

Thrombin generation is influenced by different variables like age, sex, body mass index, genetic factors, and acquired conditions [6,7]. In a previous study, the ETP values of children were found to be lower than those of adults [8]. Positive correlations were found for age versus thrombin generation parameters in calibrated automated thrombography in two recent studies [9,10]. We showed that ETP and peak levels were higher in adults as compared to infants. Thus, we suggest that ETP and peak levels, the main parameters of thrombin generation, increase gradually from infancy to adulthood. As for limitations, our adult group was not adequate for representing all ages of the adult population and there was a difference between the groups in terms of sex ratios. However, we may conclude that plasma from adults may be more procoagulant than that of infants. Our findings may confirm the presence of a regulation mechanism in the coagulation parameters throughout the course of life.

## Figures and Tables

**Table 1:**
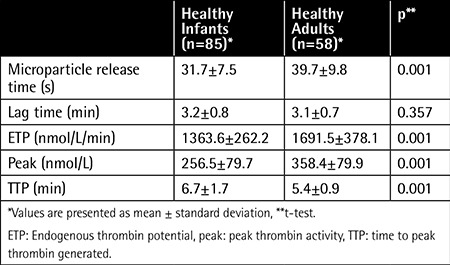
Data on indirect microparticle activity and thrombin generation parameters of the study groups.
